# High-Sensitivity cardiac Troponins in Cardio-Healthy Subjects: A Cardiovascular Magnetic Resonance Imaging Study

**DOI:** 10.1038/s41598-018-33850-9

**Published:** 2018-10-18

**Authors:** Tar-Choon Aw, Wei-ting Huang, Thu-Thao Le, Chee-Jian Pua, Briana Ang, Soon-Kieng Phua, Khung-Keong Yeo, Stuart A. Cook, Calvin W. L. Chin

**Affiliations:** 1 Department of Laboratory Medicine, Changi General Hospital, 2 Simei Street 3, Singapore, 529889 Singapore; 2 Department of Cardiology, National Heart Center Singapore, 5 Hospital Drive, Singapore, 169609 Singapore; 3 Duke-NUS Medical School, Singapore, Singapore

**Keywords:** Diagnostic markers, Myocardial infarction

## Abstract

The 99^th^ percentile upper reference limits (URL) of high-sensitivity cardiac troponin (hs-cTn) in healthy subjects are essential for diagnosis and management of cardiovascular diseases. Unless screened stringently, subclinical disease affects the derived URL. In 779 healthy subjects(49% males; 17–88 years) screened by cardiovascular magnetic resonance (CMR), the gold standard for assessing cardiac volumes and myocardial mass; and estimated glomerular filtration rate (eGFR), the 99^th^ percentile URL of hsTnT (Roche) and hs-cTnI (Abbott) were similar to the published URL. The overall 99^th^ percentile URL of hsTnT and hsTnI were 15.2 and 21.2 ng/L, respectively; males had higher values than females (hsTnT: 16.8 versus 11.9 ng/L and hsTnI: 38.8 versus 14.4 ng/L). Correlation between hsTnT and hsTnI was modest (r = 0.45; p < 0.001). A larger proportion of healthy volunteers <60 years had detectable hsTnI compared to hsTnT (n = 534; 30.0% versus 18.3%, p < 0.001). Lower eGFR was an independent clinical determinant of hsTnT, but not hsTnI. Both hs-cTn concentrations were independently associated with myocardial mass and cardiac volumes (p < 0.01 for all), but only hsTnI was independently associated with CMR multi-directional strain measures and extent of LV trabeculations (p < 0.05 for all). Differences exist between hs-cTn assays and may influence their selection depending on cardiac conditions, patient population and local factors.

## Introduction

Two high-sensitivity cardiac troponins (hs-cTn) are widely used – the Roche Diagnostics hsTnT^1^ and the Abbott Diagnostics hsTnI^2^. Hs-cTn are used for the diagnosis and risk stratification of cardiovascular disorders. In particular, myocardial infarction is diagnosed when there is clinical evidence of myocardial ischemia and a rise and/or fall in cardiac troponin concentrations with at least 1 value above the clinical decision limit defined as the 99^th^ percentile value determined from a reference population^3^. Detectable levels of hs-cTn between the assay detection limit and the URL are associated with future major adverse cardiovascular events^4,5^.

Clinical decisions are based on a key metric of 99^th^ percentile upper reference limit (URL) derived from a healthy reference population. To exclude subclinical disease, experts and practice guidelines recommend screening reference subjects with questionnaires/interviews, biomarkers (estimated glomerular filtration rate [eGFR] for renal insufficiency, natriuretic peptides for cardiac stress, and HbA1c for diabetes) and imaging^6,7^. In fact further screening with health questionnaires, eGFR, N-terminal pro–B-type natriuretic peptide (NTproBNP), and echocardiography resulted in a 50% decline in the 99th percentile URL^8,9^. In studies of community health status involving hsTnT, echocardiography^10^ and cardiac magnetic resonance (CMR) imaging^11^ revealed significant structural cardiac abnormalities.

There is great variation in the reported hs-cTn URLs (29.4% for hsTnT and 80.0% for hsTnI)^12^. This is due to inadequate sample size and varying composition, age, gender and screening procedures employed for the reference population. Over 300 subjects per gender are recommended for a sufficiently large URL study. There are few studies with adequate sample size: 7 separate hsTnI and 7 individual hsTnT URL studies^12^. Moreover, only 3 studies compared both hs-cTn together in the same population^12^ and they excluded subclinical cardiac disease using questionnaires, biomarkers (2 studies) and electrocardiogram (1 study); cardiac imaging was not done. A handful of small echocardiographic hs-cTn URL studies exist for hsTnT^8,9,13^ and a prototype hsTnI^14^.

No data is available on hs-cTn levels in those deemed cardio-healthy by the gold standard assessment of cardiac function and LV mass - cardiac magnetic resonance (CMR). All URL studies have used eGFR of <60 mL/min/1.73 m^2^ to exclude renal insufficiency and they may have inadvertently included some subjects with subclinical renal disease. Key questions remain:

- What are normal hs-cTn levels in truly cardio-healthy subjects screened by CMR?
- What is the impact on hs-cTn URLs of adopting higher eGFR values (90 mL/min/1.73 m^2^) as opposed to 60 mL/min/1.73 m^2^ to screen out subclinical renal disease?

We systematically established the 99^th^ percentile reference limits for high-sensitivity cardiac troponins T (hsTnT) and I (hsTnI) in the same cohort of well-characterized multi-ethnic Asians who were deemed cardio-healthy based on normal cardiovascular magnetic resonance (CMR) imaging^15,16^, and renal-healthy based on the eGFR^17^(>60 mL/min/1.73m^2^versus >90 mL/min/1.73 m^2^). Furthermore, we comprehensively examined clinical determinants and CMR measures of left ventricular (LV) morphology and function associated with the two high-sensitivity cardiac troponins (hsTnT and hsTnI) in these cardio-renal healthy volunteers.

## Results

On the basis of normal CMR and renal function, 779(males, n = 379 [49%]; median age52years [range: 17 to 88]) cardio-renal healthy Singaporeans were analyzed in this study. There were 209 (26.8%), 58 (7.4%) and 23 (3.0%) participants treated for hypertension, hyperlipidemia and diabetes mellitus, respectively (Table 1).

**Table 1:** Baseline Characteristics of Study Population.

	All (n = 779)	Males (n = 379)	Females (n = 400)
**Clinical Parameters**			
Age, years	51.2 ± 14.3	52.0 ± 14.9	50.4 ± 13.8
Smoking, n (%)	24 (3.1%)	20 (5.3%)	4 (1.0%)
Hypertension, n (%)	209 (26.8%)	127 (33.5%)	82 (20.5%)
Diabetes Mellitus, n (%)	23 (3.0%)	11 (2.9%)	12 (3.0%)
Hyperlipidemia, n (%)	58 (7.5%)	36 (9.5%)	22 (5.5%)
Body surface area, m^2^	1.70 ± 0.20	1.83 ± 0.17	1.59 ± 0.14
Systolic blood pressure, mmHg	135 ± 18	139 ± 16	131 ± 19
**Cardiovascular Magnetic Resonance**			
LV EF, %	64 ± 6	63 ± 6	65 ± 6
RV EF, %	62 ± 7.4	59 ± 7	64 ± 6
Indexed LV mass, g/m^2^	45 ± 10	50 ± 9	39 ± 7
Indexed LV EDV, mL/ m^2^	69 ± 10	71 ± 11	66 ± 9
Indexed LV ESV, mL/ m^2^	24 ± 7	26 ± 7	23 ± 6
Indexed RV EDV, mL/ m^2^	71 ± 13	75 ± 13	65 ± 10
Indexed RV ESV, mL/ m^2^	27 ± 9	31 ± 9	24 ± 7
Indexed LA area, cm^2^/ m^2^	11.6 ± 2.0	11.0 ± 2.0	12.1 ± 1.9
Indexed RA area, cm^2^/ m^2^	10.7 ± 2.0	11.0 ± 2.1	10.5 ± 1.8
Global FD	1.21 ± 0.03	1.22 ± 0.03	1.21 ± 0.03
Mean apical FD	1.22 ± 0.05	1.24 ± 0.05	1.21 ± 0.05
Maximum apical FD	1.28 ± 0.05	1.29 ± 0.05	1.27 ± 0.05
Global circumferential strain, %	−21.2 ± 2.9	−19.5 ± 2.0	−22.9 ± 2.6
Global radial strain, %	48.4 ± 11.0	42.4 ± 7.6	54.4 ± 10.7
Global longitudinal strain, %	−20.0 ± 2.5	−18.6 ± 2.0	−21.4 ± 2.1
**Biochemical Markers**			
Estimated GFR (mL/min/1.73 m^2^)	96.4 ± 21.1	104 ± 21	88 ± 17
NTproBNP, ng/L	29.6 [14.8, 54.9]	20.8 [9.6, 39.4]	39.1 [21.1, 65.8]
hsTnT, ng/L	2.5 [2.5, 5.5]	2.5 [2.5, 6.4]	2.5 [2.5,2.5]
hsTnI, ng/L	0.75 [0.75, 2.1]	1.5 [0.75, 2.7]	0.75 [0.75,1.5]

### Distribution and Clinical Determinants of Cardiac Troponin Concentrations

**Figure 1:**
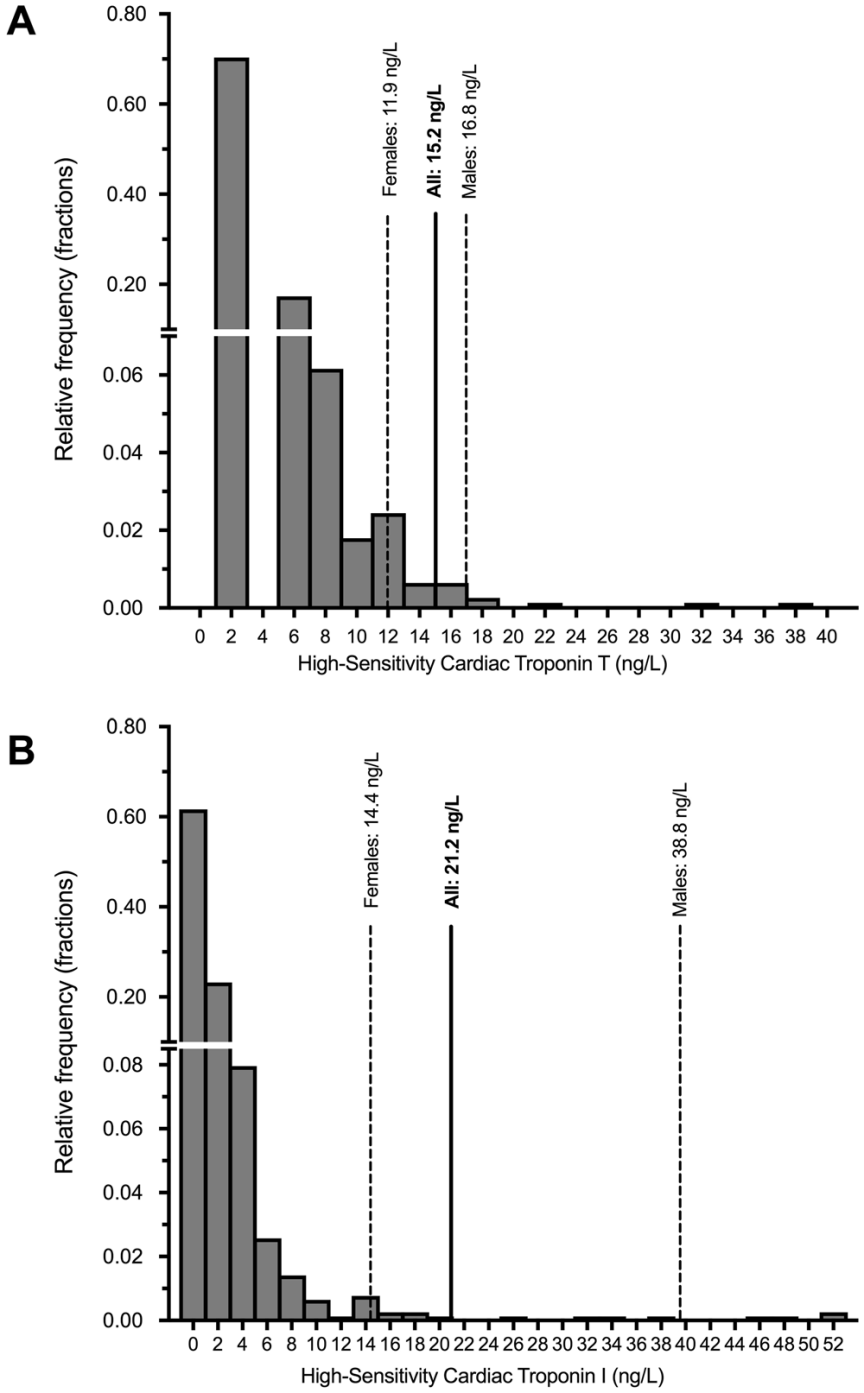
Distribution of high-sensitivity cardiac troponin T (Panel A) and I (Panel B) Concentrations in Cardio-renal Healthy Asians.

The overall and sex-stratified distributions of troponin values were non-Gaussian. The 99^th^ percentile values of hsTnT concentrations in all patients, males and females were 15.2 (90% confidence interval (CI): 13.2–18.9), 16.8 (90% CI: 15.0–38.2) and 11.9 (90% CI: 11.1–32.9) ng/L respectively. For hsTnI, the 99^th^ percentile values in all patients, males and females were 21.2 (90% CI: 14.4–37.3), 38.8 (90% CI: 18.2–51.6) and 14.4 (90% CI: 6.9–20.1) ng/L, respectively (Fig. 1).

To examine the impact of external factors on the 99^th^ percentile values for cardiac troponin in these CMR healthy subjects, we sequentially excluded 213 participants with cardiovascular risk factors and 15 participants with NTproBNP > 125 ng/L **(**Table 2**)**. Even after the pruning exercise, cardiac troponin (hsTnT and hsTnI) distributions remained non-Gaussian. The 99^th^ percentile values for both hsTnT and hsTnI in all CMR healthy patients (males and females) remained fairly similar despite exclusion of possible factors that may contribute to subclinical cardiac disease; the differences between groups were less than the relative change value for troponins. Table 2 revealed that applying an even more stringent eGFR cut-off (>90 mL/min/1.73 m^2^) for renal health would only impact hsTnT especially in men as hsTnT is more closely associated with eGFR and the number of subjects available for analysis declined to less than 300.

**Table 2:** Effect of Patient Selection on the Troponin 99^th^Percentile Upper Reference Limit.

Population	hsTnT, ng/L (90% confidence interval)			hsTnI, ng/L (90% confidence interval)		
	All	Males	Females	All	Males	Females
All (Total = 779; F = 400, M = 379)	15.2 (13.2–18.9)	16.8 (15.0–38.2)	11.9 (11.1–32.9)	21.2 (14.4–37.3)	38.8 (18.2–51.6)	14.4 (6.9–20.1)
Group 1 minus diabetes mellitus (Total = 756; F = 388, M = 368)	15.2 (13.2–18.9)	15.7 (13.9–22.3)	11.9 (11.1–32.9)	22.5 (14.5–45.5)	39.7 (18.2–51.6)	14.5 (7.4–20.1)
Group 1 minus hyperlipidemia (Total = 721; F = 378, M = 343)	15.1 (12.6–18.9)	16.0 (13.9–22.3)	12.0 (11.1–32.9)	24.6(14.5–45.5)	41.7 (18.2–51.6)	14.6 (7.4–20.1)
Group 1 minus hypertension (Total = 570; F = 318, M = 252)	13.7 (11.9–18.9)	15.8*	12.2 (11.1–32.9)	22.8 (10.2–47.8)	41.9*	14.3 (6.2–20.1)
Group 1 minus NTproBNP > 125 ng/L (Total = 744; F = 376, M = 368)	15.1(12.3–22.3)	16.9 (13.9–38.2)	11.9 (10.4–32.9)	23.1 (14.5–45.5)	39.6 (18.2–51.6)	14.4 (6.2–20.1)
Group 1 minus diabetes mellitus, hyperlipidemia, hypertension** (Total = 566; F = 316, M = 250)	13.8 (11.9–18.9)	15.8*	12.3 (11.1–32.9)	23.2 (10.3–47.8)	42.1*	14.3 (6.2–20.1)
Group 6 minus NTproBNP > 125 ng/L** (Total = 550; F = 302, M = 248)	12.9 (11.9–17.7)	15.8*	11.9 (9.0–32.9)	24.9 (10.1–47.8)	42.3*	13.8 (5.3–20.1)
Group 1 minus eGFR <90 mL/min/1.73 m^2^ (Total = 465; F = 302, M = 163)	11.9 (10.4–13.2)	12.6*	11.8 (10.4–12.3)	21.9 (9.4–51.6)	40.0*	12.8 (5.3–20.1)

*90% confidence intervals not estimated because sample size was less than 300.

**Subjects excluded are not mutually exclusive.

**Figure 2:**
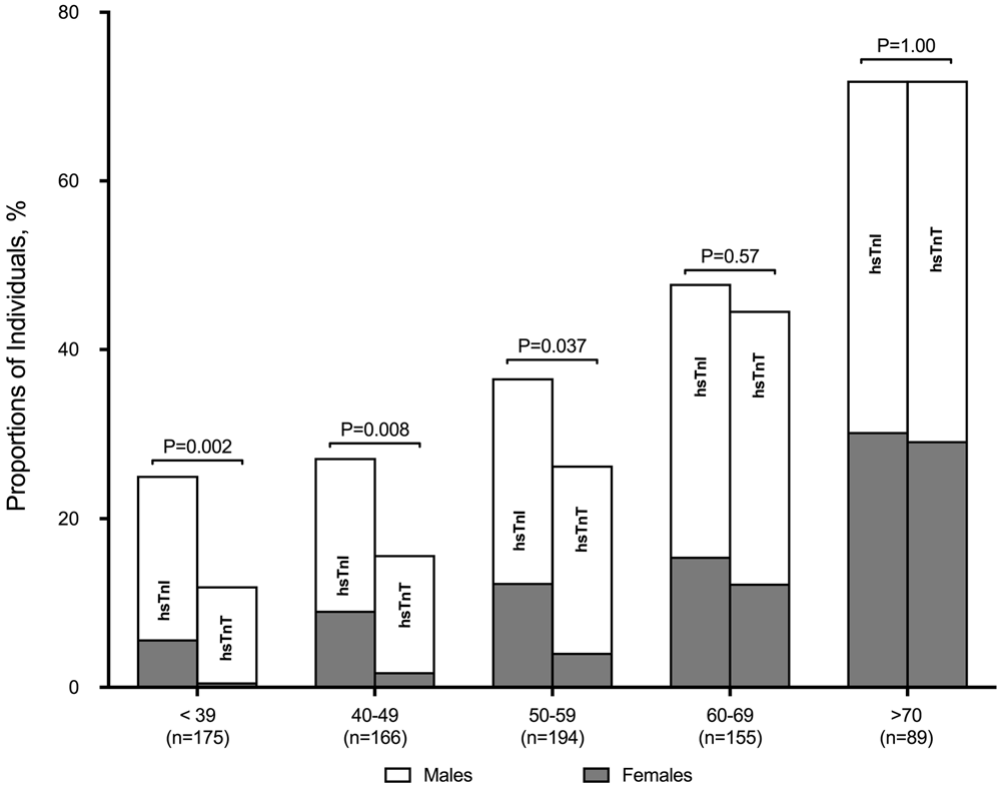
Proportion of Individuals with Detectable Cardiac Troponins.

**Figure 3:**
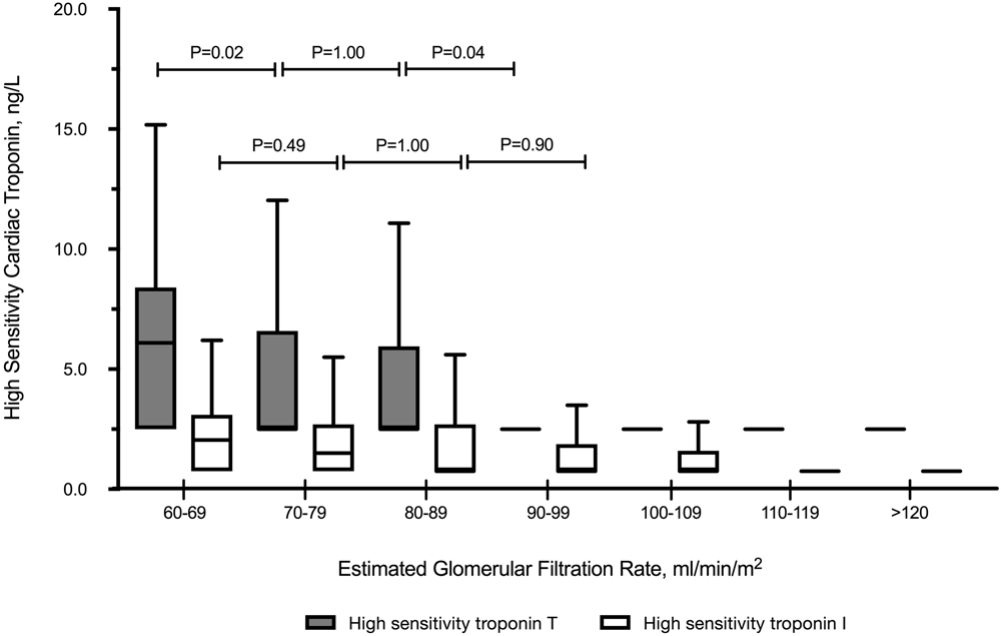
Association Between High-Sensitivity Cardiac Troponins and Renal Function. Results presented in box and whiskers (Tukey method).

The correlation between hsTnT and hsTnI was modest (r = 0.45; p < 0.001). Overall, more healthy volunteers had detectable hsTnI compared to hsTnT (38.4% versus 29.7%, respectively; p < 0.001). The proportion of healthy individuals with detectable cardiac troponin concentrations increased with age, with some differences observed between the two high-sensitivity assays. In healthy participants >60 years old (n = 244), the proportion of individuals with hsTnI and hsTnT concentrations above the LOD were similar (56.6% versus 54.6% respectively; p = 0.72). Conversely, a larger proportion of younger individuals <60 years old had detectable hsTnI compared to hsTnT (30.0% versus 18.3%, respectively; p < 0.001; Fig. 2), particularly in younger females (17.5% versus 4.2%, respectively; p < 0.001). Male sex, increasing age and systolic blood pressure were independent determinants of both hsTnT and hsTnI. Despite normal renal function (eGFR≥60 mL/min/1.73 m^2^) in all individuals, a lower eGFR was independently associated with higher hsTnT concentrations, but not with hsTnI **(**Table 3). Every 10 mL/min/1.73 m^2^ decrease in eGFR from 90 mL/min/1.73 m^2^ was significantly associated with a stepwise increase in hsTnT concentrations **(**Fig. 3).

**Table 3:** Clinical Determinants Associated with high-sensitivity Troponins.

High-sensitivity Troponin T	Univariate Unstandardized Beta (standard error)	P Value	Multivariate Unstandardized Beta (standard error)	P Value
Age, 10 years	0.14 (0.01)	<0.001	0.11 (0.01)	<0.001
SBP, 10 mmHg	0.07 (0.01)	<0.001	0.02 (0.01)	0.04
Male	0.34 (0.04)	<0.001	0.26 (0.04)	<0.001
BSA, m^2^	0.41 (0.09)	<0.001	—	
Hypertension	0.28 (0.04)	<0.001	—	
Hyperlipidemia	0.29 (0.07)	<0.001	—	
Diabetes	0.30 (0.11)	0.006	—	
Smoking	0.03 (0.11)	0.80	—	
eGFR, 10 mL/min/1.73 m^2^	−0.09 (0.01)	<0.001	−0.03 (0.01)	0.006
**High-sensitivity Troponin I**	**Univariate Unstandardized Beta (standard error)**	**P Value**	**Multivariate Unstandardized Beta (standard error)**	**P Value**
Age, 10 years	0.13 (0.02)	<0.001	0.10 (0.02)	<0.001
SBP, 10 mmHg	0.11 (0.02)	<0.001	0.06 (0.02)	<0.001
Male	0.43 (0.06)	<0.001	0.37 (0.06)	<0.001
BSA, m^2^	0.67 (0.14)	<0.001	—	
Hypertension	0.25 (0.06)	<0.001	—	
Hyperlipidemia	0.07 (0.11)	0.50	—	
Diabetes	0.10 (0.17)	0.55	—	
Smoking	0.09 (0.17)	0.57	—	
eGFR, 10 mL/min/1.73 m^2^	−0.10 (0.01)	<0.001	—

### Associations Between Cardiac Troponin Concentrations and Cardiac Morphology and Function

Log-transformed troponin (hsTnT and hsTnI) concentrations were weakly associated with LV mass (hsTnT: r = 0.26; p < 0.001 and hsTnI: r = 0.29; p < 0.001). Similar weak correlations were observed with cardiac volumes in the LV (hsTnT: r = 0.13; p < 0.001 and hsTnI: r = 0.18; p < 0.001) and RV (hsTnT: r = 0.13; p < 0.001 and hsTnI: r = 0.18; p < 0.001). Both hsTnT and hsTnI concentrations were associated with LV mass and cardiac volumes, after adjusting for age, sex, systolic blood pressure and eGFR (p < 0.01 for all).

**Figure 4:**
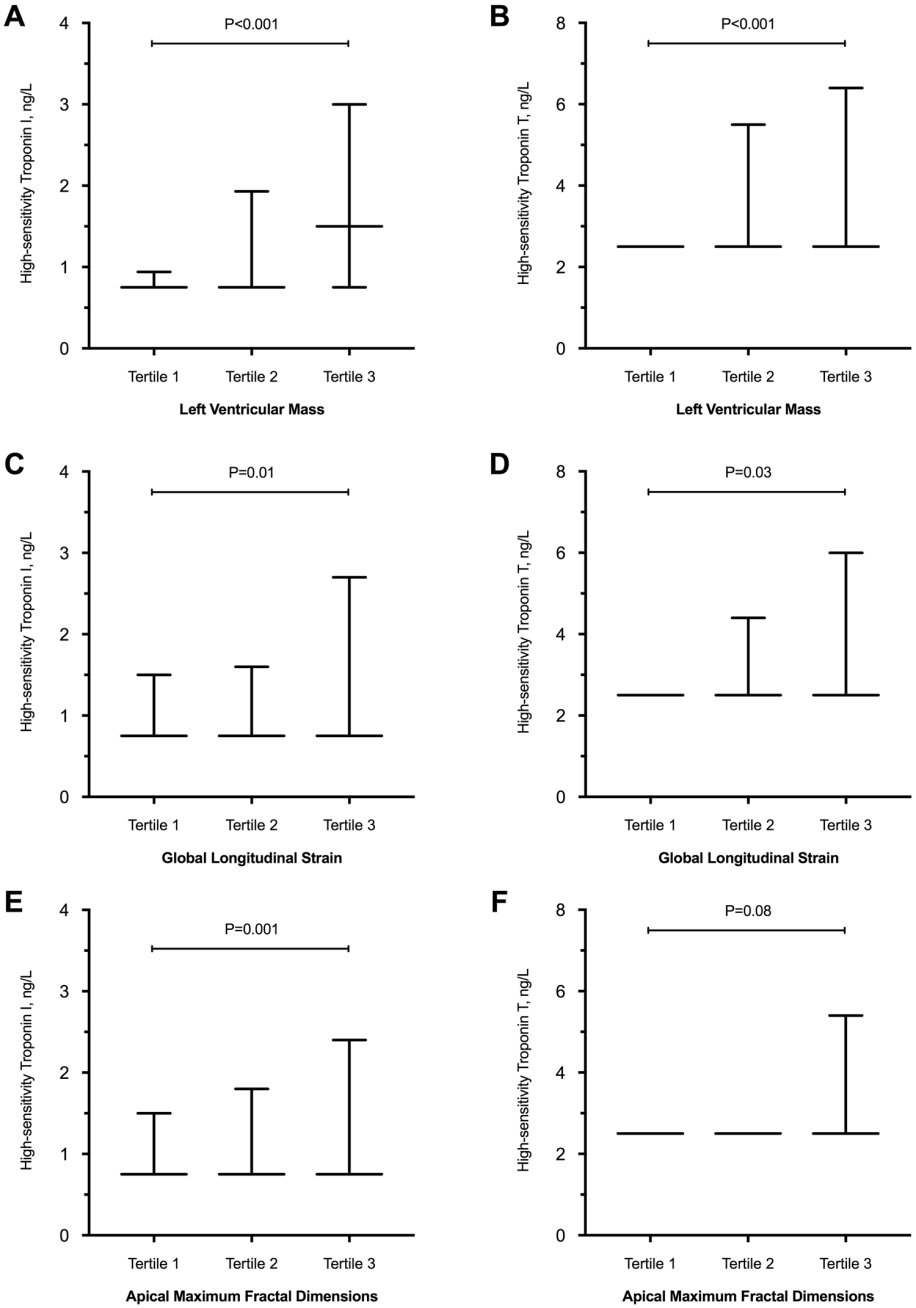
Association Between High-Sensitivity Cardiac Troponins and Left Ventricular Mass (Panels A and B), Global Longitudinal Strain (Panels C and D) and Apical Maximum Fractal Dimensions (Panels E and F). Results presented in median and interquartile range.

Cardiac troponin T and I concentrations were associated with multi-directional strain (global circumferential, radial and longitudinal). However, only hsTnI were independently associated with myocardial deformation after adjusting for age, sex, systolic blood pressure and eGFR. Similar independent associations with hsTnI were observed with FD measures (Fig. 4). Of note, hsTnT and hsTnI concentrations did not correlate with ejection fractions in the LV (hsTnT: r = −0.06; p = 0.10 and hsTnI: r = −0.02; p = 0.56) and RV (hsTnT: r = −0.02; p = 0.64 and hsTnI: r = −0.02; p = 0.67). Equally, no correlation was found between hsTnT, hsTnI and the cardiac index (r = −0.07; P = 0.04, and r = −0.01; P = 0.74, respectively).

It was perhaps not surprising that the group with both detectable hsTnI and hsTnT had more males, older subjects, more coronary artery risk factors (hypertension and hyperlipidemia) and lower eGFR. Moreover, individuals in this group demonstrated increased LV mass index, LV trabeculations and reduced multi-directional strain measures (despite all measures being within the normal range) compared to those with undetectable circulating troponins (Table 4).

**Table 4:** Clinical and CMR Characteristics of Individuals with Detectable High-Sensitivity Troponin I and/or T.

	Below LOD for both assays	Detectable hsTnI	Detectable hsTnT	Detectable hsTnI and hsTnT	P Value
	(n = 406)	(n = 142)	(n = 74)	(n = 157)	
Age, years	47 [37,56]	51 [42,60]	57 [47,64]	63 [55,73]	<0.001
Males, n (%)	126 (31)	79 (56)	55 (74)	119 (76)	<0.001
SBP, mmHg	130 ± 17	138 ± 18	137 ± 16	145 ± 18	<0.001
BSA, m^2^	1.66 ± 0.19	1.74 ± 0.20	1.78 ± 0.22	1.74 ± 0.20	<0.001
Smoking, n (%)	9 (2.2)	6 (4.2)	5 (6.7)	4 (2.5)	0.16
Hypertension, n (%)	74 (18)	34 (24)	28 (38)	73 (46)	<0.001
Diabetes Mellitus, n (%)	9 (2.2)	2 (1.4)	4 (5.4)	8 (5.1)	0.11
Hyperlipidemia, n (%)	23 (5.7)	4 (2.8)	9 (12)	22 (14)	<0.01
Estimated GFR (mL/min/1.73 m^2^)	101 [90,115]	95 [83,108]	82 [75,98]	81 [71,92]	<0.001
LV EF, %	64 ± 6	65 ± 7	63 ± 6	64 ± 7	0.06
RV EF, %	61 ± 7	62 ± 7	61 ± 8	62 ± 8	0.56
LV indexed mass, g/m^2^	42 ± 9	46 ± 10	47 ± 9	49 ± 9	<0.001
LV indexed EDV, mL/m^2^	68 ± 9	69 ± 11	69 ± 10	70 ± 12	0.31
LV indexed ESV, mL/m^2^	24 ± 6	24 ± 7	26 ± 6	26 ± 8	0.13
RV indexed EDV, mL/m^2^	70 ± 12	71 ± 13	71 ± 13	73 ± 15	0.05
RV indexed ESV, mL/m^2^	27 ± 8	27 ± 9	28 ± 10	29 ± 10	0.17
Global circumferential strain, %	−21.7 [−23.9,−20.1]	−20.4 [−22.9,−18.4]	−19.6 [−21.1,−19.0]	−18.9 [−20.9,−17.3]	<0.01
Global radial strain, %	48.8 [42.2,57.2]	44.6 [39.7,52.1]	43.8 [38.8,48.8]	41.1 [35.4,54.8]	0.02
Global longitudinal strain, %	−20.7 [−22.3,−19.0]	−19.2 [−21.5,−17.1]	−19.0 [−20.7,−17.5]	−18.9 [−19.9,−16.0]	<0.01
Global FD	1.21 [1.19,1.23]	1.22 [1.19,1.24]	1.21 [1.18,1.24]	1.22 [1.19,1.25]	0.03
Mean apical FD	1.22 [1.18,1.25]	1.23 [1.19,1.27]	1.23 [1.19,1.26]	1.24 [1.20,1.27]	<0.01
Maximum apical FD	1.28 [1.24,1.31]	1.29 [1.26,1.33]	1.28 [1.24,1.34]	1.30 [1.26,1.33]	<0.01

## Discussion

Using a very comprehensive approach to define cardio-renal health (including CMR imaging) we have established overall and sex-specific 99^th^ percentile troponin concentrations in the same Asian population of sufficient size for the two hs-cTn assays, Roche hsTnT and Abbott hsTnI. The overall hs-cTn URLs in our cardio-healthy subjects are 15.2 ng/L for hsTnT and 21.2 ng/L for hsTnI. These values are not dissimilar from that previously reported of 14 ng/L (hsTnT) and 26 ng/L (hsTnI)^12^. Male gender and increasing age were independent determinants in both assays. Both hsTnT and hsTnI concentrations were independently associated with myocardial mass and cardiac volumes after adjusting for age, sex, systolic blood pressure and eGFR (p < 0.01 for all). There were notable differences between the two high-sensitivity assays. Only hsTnI was independently associated with myocardial multi-directional strain and LV trabeculations, an increasingly recognised marker of cardiac remodelling. HsTnI assay had increased sensitivity for detecting measurable troponin values (> LOD) in younger individuals (particularly women) compared to hsTnT. A lower eGFR in this cardio-renal healthy population was significantly associated with higher hsTnT, but not hsTnI.

Currently, the clinical decision limit in the diagnosis of myocardial infarction is based on the 99^th^ percentile cardiac troponin values derived from a normal, healthy population^18^. However, there is insufficient guidance on what constitutes a “normal and healthy” population^19^.The 99^th^ percentile value depends on the characteristics of the reference population. Not surprisingly, applying more stringent selection criteria reduces the number of outliers and lowers the 99^th^ percentile values due in part to younger subjects remaining in the selected group^8^. In our study of normal CMR subjects the 99^th^ percentile values remained unchanged or altered minimally after pruning with clinical history (treatment for diabetes, hyperlipidemia, and hypertension) and biomarkers (eGFR and NTproBNP). Thus for the purpose of deriving hs-cTn URL in this cohort, history and biomarkers provided minor additional signal to CMR in screening out subclinical cardiac disease. The observed difference in URL with or without pruning was less than the reported reference change values (RCV)^20^ for hsTnI (49–69%) and hsTnT (23–32%). The URL of hs-cTn will continue to be influenced by the distortionary effects of the highest few troponin values when each pruning modality is applied. Unless pruning measures eliminate these high values, the URL will remain relatively unchanged. However, our 99^th^ percentile URL troponin values were indeed lower compared to other recent Asian studies^12^. It is noteworthy that using such extensive screening methodologies comes at a cost that may not feasible in many other populations. Instead, a more pragmatic and standardized definition of a reference population would be preferred to harmonise comparison of assays and values across studies.

The second issue is that of sample size. A sufficiently large sample size is needed to minimise the effects of extreme values on the 99^th^ percentile values. To achieve a tolerance level of 0.95, a sample size of 600 (300 males and 300 females)would be necessary to determine the 99^th^ percentile values for either sex^21^. With these issues in mind, we used CMR to assess cardiovascular health^22^ and ensured that it was sufficiently powered to derive sex-specific 99^th^ percentile values for both hsTnT and hsTnI.

Consequent to our stringent patient selection guided by CMR and the large number of young healthy volunteers (44% of the healthy participants were less than 50 years old), the proportion of patients with detectable troponins by either assay was expectedly low. Our findings suggest that hsTnI had significantly higher sensitivity for detectable cardiac troponin values in younger individuals, particularly females, compared to hsTnT.

Previous studies comparing hsTnT and hsTnI had demonstrated a stronger association between eGFR and hsTnT in patients with chronic kidney disease^23,24^. Our study extends these findings to a well-characterised cardio-healthy population with normal renal function. Despite normal renal function (eGFR ≥60 mL/min/1.73 m^2^), eGFR was an independent determinant of hsTnT. For every 10 mL/min/1.73 m^2^ decrease in eGFR from 90 mL/min/1.73 m^2^, there was a significant and step-wise increase in hsTnT concentrations. The mechanisms for the increased variability of hsTnT with renal function remain unclear, although it has been postulated that hsTnT fragments (<18 kDa) present in chronic renal disease can cross-react with current clinical hsTnT assays^25,26^. Adopting a tighter eGFR cutoff (>90 mL/min/1.73 m^2^) to rule out even mild cases of renal insufficiency in URL studies will impact hsTnT more than hsTnI as well as crimp the size of the renal-healthy cohort especially amongst men. This is borne out in our data (see Table 2); 15.5% (85/550) of the healthy men were eliminated with a consequent reduction of the male hsTnT URL from 15.8 ng/L to 12.6 ng/L and the hsTnI from 42.3 ng/L to 40.0 ng/L. Such stringency will render URL studies even more onerous. We agree with the expert committee Practice Guidelines^7^ for the modest eGFR cutoff of 60 mL/min/1.73 m^2^.

To date, elevated cardiac troponin has been considered the *sine qua non* for myocardial infarction. Recently, we have demonstrated an association between cardiac troponins and hypertrophic response in two common causes of heart failure: calcific aortic stenosis^27^ and hypertensive heart disease^28^. In separate studies, cardiac troponins were independently associated with increased LV mass and myocardial fibrosis on CMR, supporting the hypothesis that the release of cardiac troponin relates to the myocardial injury that accompanies increased myocardial hypertrophy and fibrosis. The current study extends the association between cardiac troponins and cardiac remodelling to healthy volunteers, albeit with weaker correlations. Of note, hsTnI was independently associated with sensitive CMR measures of intrinsic cardiac function: multi-directional strain and LV trabeculations. These findings suggest that hsTnI has higher clinical sensitivity (ability to detect low troponin concentrations in younger individuals and subtle changes in cardiac function) and more cardiac specificity (less influenced by other clinical confounders such as renal function) than hsTnT.

Cardiac troponin concentrations correlate with LV mass and females have less LV mass than males^15,29^. It is perhaps not surprising that such biological differences contribute to lower 99^th^ percentile cardiac troponin concentrations in females compared to males, as also demonstrated in our study. The use of sex-specific troponin thresholds has already been recommended by the ESC/ACCF/AHA/WHF Task Force for the Universal Definition of Myocardial Infarction^18^ and the American Association for Clinical Chemistry/International Federation of Clinical Chemistry and Laboratory Medicine Task Force on Clinical Applications of Cardiac Biomarkers^7^. However, the current clinical evidence supporting the use of sex-specific thresholds are controversial^30–33^. There are relevant concerns regarding the use of sex-specific thresholds^34,35^. It is also conceivable that the clinical decision limits for myocardial infarction in different assays are not biologically equivalent^36^.

Occult underlying cardiac disease has been excluded to the greatest extent possible with CMR. We demonstrated a modest correlation between the two assays (r = 0.45; p < 0.001). Both biological and analytical characteristics of the assays may explain the observed differences between hsTnT and hsTnI^37^. Although small, these potentially important differences between troponin assays have diagnostic and prognostic impact. In myocardial infarction, the diagnostic performance of hsTnI at admission was superior to hsTnT in a subgroup of early presenters; and hsTnT was superior to hsTnI in predicting long-term prognosis^38,39^. As sensitivity of cardiac troponin assays improve, there is increasing recognition that troponin release is a continuum between health and risk^40^, an observation also supported by our current and previous work. There is increasing interest in the application of cardiac troponins in cardiovascular disorders other than myocardial infarction and its prognostic value beyond dichotomous clinical decision limits^41^. Undoubtedly, an understanding of the differences in troponin assays is crucial to guide the design of studies, interpretation of results and improvement in their clinical use.

We found the distribution of serum troponin levels remain non-Gaussian and skewed to the right. This finding has been observed even with the most sensitive hsTnI assay (Singulex; LOD of 0.091 ng/L), where distribution was highly skewed to the right in a large reference range study (n = 1,645) pre-screened with 12 biomarkers^42^. This precludes the notion that the distribution of cardiac troponin in cardio-healthy subjects tends toward Gaussian and may be amenable to a 97.5^th^ percentile upper reference limit, like other laboratory analytes^40^.

The hsTnT (Roche) has been criticised for not being a true high-sensitivity assay^43^. As demonstrated in our study, both hsTnT and hsTnI are not detectable in >50% of CMR cardio-renal healthy subjects. Does the criterion of a high-sensitivity troponin assay (defined as detectable troponin in at least 50% of healthy individuals) need revision given that even in very healthy subjects (such as our cohort) neither hsTnT nor hsTnI satisfied this criteria except perhaps hsTnI in men. In fact, when first mooted as a metric for high-sensitivity troponins^44^, none of the available troponin assays then were detectable in > 25% of healthy subjects. Larger studies and consensus statements are sorely needed in this regard.

This study has several limitations. We did not assess age-specific 99^th^ percentile cardiac troponin values. Extending the study to include each age-decile by sex would require 3,000 individuals, a prohibitive undertaking. Moreover, as highlighted in the study, current troponin assays have limited sensitivity in younger individuals and thus will not likely yield meaningful results in the younger population and thus mitigate against their inclusion in the reference population. Lastly, it is possible that some healthy volunteers have subclinical myocardial ischemia or coronary atherosclerosis. Of note, we had previously reported hsTnI concentrations were similar between patients with and without coronary artery disease and no correlation was observed between coronary calcium scores and hsTnI^27^. This supports the observation that myocardial ischemia and/or coronary atherosclerosis probably have less effects on troponin elevations than myocardial structural changes (elevated mass, necrosis and fibrosis).

In conclusion, this study affirms that males have significantly higher troponin concentrations compared to females. Whether sex-specific troponin concentrations should be considered in the diagnosis of myocardial infarction requires further prospective validation. In a highly enriched healthy population, our study demonstrated differences between hsTnT and hsTnI that may influence the selection of assays depending on the cardiac condition and patient population.

## Methods

### Study Population

Healthy Singaporeans without symptoms, clinical or family history of cardio- and cerebrovascular diseases were prospectively recruited in an on-going bio-banking project at the National Heart Research Institute Singapore, National Heart Center Singapore to identify novel genetic variants in Asians. Renal function of all the patients was assessed using Chronic Kidney Disease Epidemiology Collaboration formula^17^. Children, subjects under 18 years old, pregnant women, and participants with estimated glomerular filtration rate (eGFR) of < 60 mL/min/1.73 m^2^ were excluded from the analysis. The study was conducted in accordance with the Declaration of Helsinki and approved by the SingHealth Centralised Institutional Review Board. Informed consent was taken from all patients.

### Cardiovascular Magnetic Resonance and Image Analysis

Cardiac phenotyping using cardiovascular magnetic resonance (CMR) was performed in all participants (3 T Philips Ingenia or 1.5 T Siemens Aera). Conventional balanced steady-state free precision cine images of the vertical and horizontal long-axis planes and the sagittal LV outflow tract view were acquired. Short-axis cines were obtained from the mitral valve annulus to the apex (1.6–1.9 mm × 1.3–1.8 mm × 8 mm slice thickness; 2 mm gap). In each view, there were 30 phases per cardiac cycle.

LV mass, cardiac volumes, and function were assessed in all patients using standardized protocols (CMR42, Circle Cardiovascular imaging Inc., Calgary, Canada) as detailed previously^15^. Individuals with abnormal cardiac findings that suggest cardiomyopathies and ischemic or valvular heart diseases were excluded.

We have recently developed and published a semi-automated fractal analysis tool to assess the extent of LV trabeculations, an increasingly recognised indicator of cardiac remodelling^16^. Fractal dimensions (FD), a dimensionless measure of trabeculation complexity, were measured using the LV short axis cine images at end-diastole. As each slice is a two-dimensional plane, the range of FD is between 1 and 2. Global, mean apical and maximum apical FD values were derived.

In a sub-set of healthy volunteers, multi-directional strain (peak global longitudinal, circumferential and radial) was assessed using the Tissue Tracking Plugin in CMR42. Peak circumferential and peak radial strains were measured from the LV short axis cine images; peak longitudinal strain was measured from the vertical and horizontal long axis cine images. We had previously tested and reported excellent inter- and intra-observer variability of tissue tracking to measure multi-directional strain^16^.

### Measurements of Serum Cardiac Troponins

Serum samples were collected from participants on the day of CMR and initially frozen at −70°. Biochemical analyses were performed in a single freeze-thaw cycle over 4 assay runs with the same lot of reagents in a laboratory accredited by the College of American Pathologists (Changi General Hospital, Singapore).

Serum hsTnT (STAT; Roche Diagnostics, Pensberg, Germany) was analysed using electro-chemiluminescence on the Cobas E602 immunoassay analyzer (Roche Diagnostics Asia-Pacific, Singapore). We have recently demonstrated the limit of detection (LOD) and the concentration at the 10% inter-assay coefficient of variation (CV) for hsTnT was 5 ng/L and 11.5 ng/L, respectively; and the between-day precision was < 3%^45^. Serum hsTnI (ARCHITECT STAT High-sensitive Troponin-I; Abbott Diagnostics, Abbott Park, IL) was determined using chemiluminescent microparticle immunoassay on the ARCHITECT i2000SR analyzer (Abbott Diagnostics, Singapore). In our recent study^46^, the LOD for hsTnI was 1.5 ng/L, the concentration at 10% inter-assay CV was 6.0 ng/L and the between-day precision was < 5%. Serum NTproBNP (proBNP II STAT; Roche Diagnostics, Pensberg, Germany) was assayed using electro-chemiluminescence (Cobas E602 analyzer, Roche Diagnostics Asia-Pacific, Singapore). The manufacturer-reported LOD for NTproBNP was 5 ng/L. Biomarker concentrations less than the detection levels were assigned a value equivalent to half the LOD.

### Statistical Analysis

Recent publications^47,48^ have shown that the nonparametric approach in combination with a conservative treatment of outliers is the preferred method for determination of the 99^th^percentile URL for hs-cTn. The 99^th^ percentile values were determined by 1-tailed non-parametric statistics according to CLSI guidelines^49–51^ with no exclusion of outliers. Continuous variables were assessed for normal distribution using the Shapiro-Wilk test. Data were presented in either mean ± SD or median [interquartile range], as appropriate. Depending on data distribution, parametric Student t test and 1-way ANOVA or the non-parametric Mann-Whitney U and Kruskal-Wallis tests were used to compare groups of continuous variables. Categorical variables were compared using the χ^2^ test. Multi-variable linear regression models were used to establish clinical determinants associated with cardiac troponins: clinically relevant variables that demonstrated univariate association with cardiac troponins (p < 0.05) were selected in the multi-variable linear regression models (forward method). The associations between cardiac troponins and CMR measures of LV mass, fractal dimensions (FD), cardiac volumes and function were examined using multi-variable linear regression, adjusting for potential clinical confounders. Log-transformed troponin (hsTnT and hsTnI) concentrations were used in the analyses because of non-normal distribution. All statistical analyses were performed using MedCalc 18.0 (MedCalc Software, Ostend, Belgium) and Stata Release 14.0 (StataCorp., Texas, USA). Statistical significance was taken as a 2-sided p < 0.05.

## Data Availability

The datasets generated during and/or analysed during the current study are not publicly available due to privacy issues and national laws but are available from the corresponding author on reasonable request under the provision that data may not leave the hospital/center premises.
