# Author Correction: High-Sensitivity cardiac Troponins in Cardio-Healthy Subjects: A Cardiovascular Magnetic Resonance Imaging Study

**DOI:** 10.1038/s41598-019-42587-y

**Published:** 2019-05-22

**Authors:** Tar-Choon Aw, Wei-ting Huang, Thu-Thao Le, Chee-Jian Pua, Briana Ang, Soon-Kieng Phua, Khung-Keong Yeo, Stuart A. Cook, Calvin W. L. Chin

**Affiliations:** 1 Department of Laboratory Medicine, Changi General Hospital, 2 Simei Street 3, Singapore, 529889 Singapore; 2 Department of Cardiology, National Heart Center Singapore, 5 Hospital Drive, Singapore, 169609 Singapore; 3 Duke-NUS Medical School, Singapore, Singapore

Correction to: *Scientific Reports* 10.1038/s41598-018-33850-9, published online 18 October 2018

The original version of this Article contained errors in the title of the paper, where “High-Sensitivity cardiac Troponins in” was incorrectly given as “High-Sensitivitycardiac Troponinsin”. This has now been corrected in the PDF and HTML versions of the Article.

